# The Same Synaptic Vesicles Originate Synchronous and Asynchronous Transmitter Release

**Published:** 2015

**Authors:** P. N. Grigoryev, A. L. Zefirov

**Affiliations:** Kazan State Medical University, Ministry of Health of the Russian Federation, Butlerov Str., 49, Kazan, 420012, Russia

**Keywords:** motor nerve ending, evoked synchronous and asynchronous transmitter release, Ca2+, Sr2+, Ba2+ ions, synaptic vesicle exocytosis and endocytosis, synaptic vesicle pools

## Abstract

Transmitter release and synaptic vesicle exo- and endocytosis during high-frequency stimulation (20 pulses/s) in the extracellular presence of different bivalent cations (Ca^2+^, Sr2+ or Ba2+) were studied in frog cutaneous pectoris nerve-muscle preparations. It was shown in electrophysiological experiments that almost only synchronous transmitter release was registered in a Ca^2+^-containing solution; a high intensity of both synchronous and asynchronous transmitter release was registered in a Sr2+-containing solution, and asynchronous transmitter release almost only was observed in a Ba2+-containing solution. It was shown in experiments with a FM 1-43 fluorescent dye that the synaptic vesicles that undergo exocytosis-endocytosis during synchronous transmitter release (Ca-solutions) are able to participate in asynchronous exocytosis in Ba-solutions. The vesicles that had participated in the asynchronous transmitter release (Ba-solutions) could subsequently participate in a synchronous release (Ca-solutions). It was shown in experiments with isolated staining of recycling and reserve synaptic vesicle pools that both types of evoked transmitter release originate from the same synaptic vesicle pool. ;

## INTRODUCTION

Transmitter release in chemical synapses involves the release of discrete packages of transmitter (quanta) through fusion of a vesicle with the presynaptic membrane. The fusion process may occur either at rest (spontaneous transmitter release) or after the action potential reaches the presynaptic terminal and voltage- gated calcium channels-mediated influx of Ca^2+^into nerve endings occurs (evoked release). The evoked transmitter release is caused by two components: synchronous, where transmitter quanta are released within several milliseconds after an action potential; and asynchronous release, persisting for tens or hundreds of milliseconds [1-3]. Synchronous transmitter release is the main component in most synapses, whereby more than 90% of quanta can be released at low-frequency stimulation [4, 5]. However, the share of asynchronous transmitter release rises at higher stimulation frequencies [6]. The use of solutions containing various alkaline earth metal ions is an experimental approach in changing the share of synchronous and asynchronous transmitter release. The share of asynchronous release rises when Ca^2+^ ions are replaced with Sr^2+^and Ba^2+^ [2, 7, 8]. Synchronization of the transmitter quantum release is believed to be caused by several presynaptic mechanisms, such as rapid short-term opening of calcium channels during membrane depolarization, the properties of the protein “machine” of transmitter release triggering quantal transmitter release only at high intracellular calcium concentrations, and also the short distance between the calcium channels and the calcium sensor of exocytosis [9]. Mechanisms of asynchronous neurotransmitter release remain poorly understood. The Ca^2+^ sensor of asynchronous release is thought to be located at a larger distance from the calcium channel and is characterized by different Ca^2+^binding dynamics [10-13].

Meanwhile, it can be assumed that the vesicles involved in the synchronous and asynchronous transmitter release differ from each other and can reside in independent populations. This idea is not unreasonable. First, the study of synaptic vesicle exo- and endocytosis in motor nerve endings has made it possible to identify two functionally distinct pools (recycling and reserve synaptic vesicle pools). The recycling pool is characterized by docked vesicles at active zones. This pool is quickly depleted at high-frequency activity, and its recovery is provided by vesicle mobilization and fast endocytosis. The reserve pool of synaptic vesicles is larger and participates in the replenishment of the recycling vesicle pool under high-frequency stimulation; it is involved in the release process later and is replenished by slow endocytosis [14, 15]. Second, there is evidence to suggest the existence of separate populations of vesicles ensuring spontaneous [16, 17] and asynchronous transmitter release [18, 19], which are, however, not involved in the evoked synchronous transmitter release. In this paper, we made an attempt to evaluate the identity of the vesicle pools involved in the synchronous and asynchronous transmitter release in motor nerve endings using electrophysiological approaches and confocal fluorescence microscopy.

## MATERIALS AND METHODS

**Object of study, solutions**

The experiments were performed using isolated frog cutaneous pectoris nerve-muscle preparations (*Rana temporaria*) in winter (December through February). This study was carried out in compliance with the International Guidelines for Proper Conduct of Animal Experiments. The standard Ringer’s solution was used: 115.0 mM NaCl, 2.5 mM KCl, 1.8 mM CaCl_2_, and 2.4 mM NaHCO_3_; pH 7.2–7.4 and temperature of 20°C were maintained. All the experiments were conducted only for the nerve terminals on the surface. Along with the standard solution (Ca-solution), we used solutions in which CaCl_2_ was replaced with either SrCl_2_ or BaCl_2_ at a concentration of 1.8 mM (Sr- and Ba-solutions). The evoked transmitter release and vesicle exocytosis were induced by prolonged high-frequency stimulation (20 pulses/s) of the motor nerve with square-wave electrical pulses of 0.1–0.2 ms duration at suprathreshold amplitude delivered by a DS3 stimulator (Digitimer Ltd., UK). Muscle fiber contractions were blocked by transverse muscle cutting. All the reagents were purchased from Sigma (USA).

**Electrophysiology**

Multiquantal end-plate potentials (EPPs) and monoquantal asynchronous signals were registered using glass microelectrodes (tip diameter less than 1 μm; resistance, 2–10 MΩ) filled with a 3 M KCl solution. A microelectrode was inserted into the muscle fiber at nerve endings under visual control. The resting membrane potential was monitored with a milli-voltmeter. The experiments in which the resting membrane potential decreased were discarded. The signals were converted into digital signals using ADC La-2USB. An original software Elph (developed by A.V. Zakharov) was used for signal accumulation and analysis.

**Quantitative assessment of synchronous transmitter release**

We used the modified method of variation of parameters, which was described in detail previously, for quantitative assessment of the quantal contents of EPPs [20]. The area of each EPP in the series were calculated. Thereafter, the region where the mean EPP area remained virtually unchanged was searched for on the plots showing the dynamics of EPP area decrease under high-frequency stimulation (usually stimulation for 10–30 s). EPP area variations were used to calculate the quantal value; i.e., the mean EPP area induced by one quantum of transmitter (q):

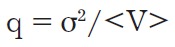



where σ is the dispersion of the EPP area and < V > is the mean EPP area in this region.

The quantal content of each EPP in the series can then be determined:

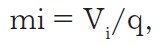



where mi is the quantal content of the i^th^EPP and Vi is the area of the i^th^EPP.

**Quantitative assessment of asynchronous transmitter release in Ca- and Sr-containing solutions**

Asynchronous transmitter release was assessed by quantifying the number of monoquantal signals appearing after EPP between stimulations (50 ms) and counting their frequencies (the number of quanta per second). Monoquantal signals were determined both automatically and visually.

**Quantitative assessment of asynchronous transmitter release in Ba-containing solutions**

Stimulation in Ba-containing solutions causes a large amount of transmitter quanta release, thus leading to stable end-plate depolarization, which is confirmed by biochemical methods [21]. High-frequency stimulation in Ba-solutions leads to an enormous amount of asynchronously occurring monoquantal signals, which overlap and cannot be determined [7, 22]. Therefore, transmitter release (frequency of monoquantal asynchronous potentials) was assessed from the depolarization change in the membrane potential mediated by asynchronous signals; correction for nonlinear summation was applied using the formula [22]:

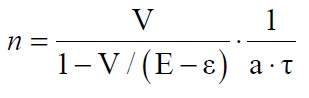



where V is depolarization of the postsynaptic membrane, mV; E is the resting membrane potential, mV; a is the mean amplitude of asynchronous monoquantal signals, mV;τ is the time constant of asynchronous monoquantal signals, ms; and ε is the acetylcholine equilibrium potential (≈ –15 mV).

**Fluorescence microscopy**

Synaptic vesicle exo- and endocytosis were examined using a FM 1-43 fluorescent dye (SynaptoGreen C4, Sigma, USA) at a concentration of 6 μM. The dye was reversibly bound to the presynaptic membrane and became trapped inside the newly formed synaptic vesicles (loading of the nerve endings) during endocytosis (after stimulation of exocytosis) [23]. Since endocytosis continues for some time after exocytosis, the dye was present in the solution both during stimulation (20 pulses/s) and within five minutes after stimulation termination. The preparation was then washed in a dye-free solution for 20 min to remove the dye bound to superficial membranes. In this case, bright fluorescent spots were observed in the nerve endings showing the accumulation of FM1-43-labeled vesicles at the active zones. Stimulation of exocytosis of the preliminarily loaded vesicles causes the release (unloading) of the dye from nerve endings [23]. Fluorescence was observed using a BX51W1 motorized microscope (Olympus, Germany) equipped with a DSU confocal scanning disk and a OrcaR2 CCD camera (Hamamatsu, Japan) connected to a PC through an Olympus Cell^P software. The optics for analyzing FM 1-43 fluorescence included Olympus U-MNB2 filters and Olympus LUMPLFL 60xw (1.0 NA) water immersion lens. Fluorescence intensity was analyzed in the 20-μm long central portion of the nerve ending. ImagePro software was used to assess the fluorescence intensity as relative fluorescence units of a pixel minus the background fluorescence. The background fluorescence was determined as the mean fluorescence intensity in a 50 × 50 pixel square in an image area showing no nerve terminals [12, 24, 25]. The profile of fluorescence in the nerve endings was calculated as the averaged fluorescence intensity of pixel rows arranged perpendicular to the longitudinal axis of the nerve ending with 1 pixel increments.

Statistical analysis was performed using the Origin Pro program. The quantitative results of the study are presented as a mean ± standard error, where *n *is the number of independent experiments. Statistical significance was assessed using Student’s t-test and ANOVA.

## RESULTS

**Transmitter release under high-frequency stimulation in Ca-, Sr-, and Ba-containing solutions**

**Fig. 1:**
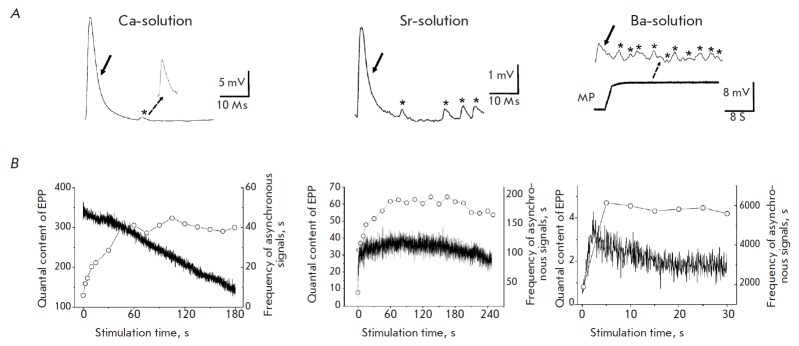
Synchronous and asynchronous transmitter release in Ca-, Sr-, and Ba- solutions under high-frequency stimulation. Examples of registered end-plate potentials (EPP) and asynchronous monoquantal signals during high-frequency stimulation (20 pulses/s) in Ca-, Sr-, and Ba-solutions (1.8 mM). The arrows indicate EPPs, and the asterisks show asynchronous monoquantal signals. It is noticeable that muscle fiber depolarization develops in Ba-solutions (*A*). Dynamics of the averaged (over all experiments) quantal content of EPP (dark lines, Y axis is shown to the left) and the averaged frequency of asynchronous monoquantal signals (white circles, Y axis is shown to the right) under high-frequency stimulation (*B*)

Multiquantal EPPs (synchronous transmitter release) accompanied by occasional monoquantal asynchronous signals were registered under high-frequency stimulation in a Ca-containing solution (*Fig. 1A*). The quantal content of the first multiquantal EPP in the series was 321 ± 120 quanta (*n *= 6),while a reduction in the quantal content was observed under high-frequency stimulation, comprising 44.3 ± 9.0% (*n *= 6) of the baseline level by the end of the third minute of stimulation (*Fig. 1B*). Asynchronous release during the first second of high-frequency stimulation was low (5.9 ± 1.4 quanta s^-1^, *n *= 7), but it rose to 40.0 ± 9.7 s^-1^ (*n *= 7) by the end of the third minute of stimulation (*Fig. 1B*). Monoquantal asynchronous signals disappeared within one second after the end of stimulation. Calculations showed that 880,251 ± 275,892 quanta (*n *= 6) were released over three minutes of stimulation in a Ca-solution through synchronous secretion, and 6751 ± 1476 quanta (n = 7) were induced by asynchronous release.

Multiquantal EPPs followed by monoquantal asynchronous signals were also recorded in Sr-solutions under high-frequency stimulation (*Fig. 1A*). The initial quantal content was significantly lower than that in a Ca-solution: i.e., 4.7 ± 0.8 (*n *= 4). By the end of the first minute of stimulation the quantal content of EPP increased to 34.3 ± 9.1 (n = 4) and remained at a relatively stable level until the end of the stimulation (*Fig. 1B*). The asynchronous release was found to be more significant in a Sr-solution than in a Ca-solution. The frequency of monoquantal signals during the first second of stimulation was 32.6 ± 5.8 quanta/s, while being 185.2 ± 10.7 quanta/s (*n *= 5) at the end of the third minute (*Fig. 1B*). After the end of stimulation, monoquantal asynchronous signals disappeared within one second. It was found that for 3 min of stimulation in a Sr-solution the amount of transmitter quanta released by the synchronous and asynchronous release was 126,359 ± 29,687 (*n *= 4) and 31,633 ± 1912 (*n *= 5), respectively. The asynchronous transmitter release appearing in Ca- and Sr-solutions did not change the resting membrane potential.

High-frequency stimulation of the motor nerve in a Ba-solution caused the emergence of one-to-threequantal EPPs and a large number of monoquantal asynchronous signals. The quantal content of EPPs grew quickly by 2–3 s and gradually decreased to 1.85 ± 0.47 (*n *= 7) by the end of stimulation (*Fig. 1B*). Prolonged stimulation during the first few seconds resulted in depolarization of muscle fibers from the resting level of –45 ± 2.9 mV to –37 ± 3.1 mV (*n *= 7) that was retained during the entire stimulation period. After the end of stimulation, the membrane potential returned to its original level within 3–7 s. Calculation using equation 3 (see the Materials and Methods section) led to a conclusion that this depolarization can be caused by asynchronous monoquantal signals with a frequency of 6131 ± 455 s^-1^ (*n *= 7). Further calculations showed that stimulation in a Ba-solution for 30 s leads to synchronous release of 1224 ± 180 quanta (*n *= 7) and an asynchronous release of 189,648 ± 41,712 quanta (n= 7).

These data suggest that almost all transmitter quanta are released synchronously (about 99.2%) in Ca-solutions under high-frequency stimulation, while asynchronous release is negligible. Application of a Srsolution reduced the share of synchronously released quanta to 80% and that of asynchronously released quanta rose to 20%. Almost only asynchronous transmitter release (approximately 99.4%) was observed in Ba-solutions. We later used Ca- and Ba-solutions to study the exo- and endocytosis of the synaptic vesicles involved in the synchronous and asynchronous transmitter release.

**Exocytosis and endocytosis of synaptic vesicles involved in synchronous and asynchronous transmitter release**

**Fig. 2:**
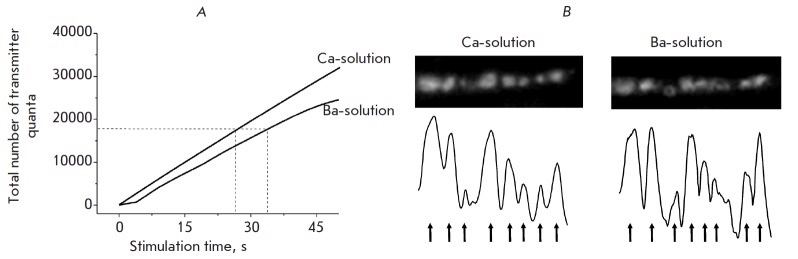
Synaptic vesicle endocytosis in Ca- and Ba- solutions. Cumulative curves of synchronously and asynchronously released quanta of neurotransmitter in Ca- and Ba-solutions during high-frequency stimulation (based on the data in *Fig. 1B*). It is noticeable that 180,000 neurotransmitter quanta (the dashed line) are released during stimulation for 30 s (A). Images of FM 1-43 fluorescence at the same nerve terminal area after loading with FM 1-43 under stimulation for 30 s in Ca- and Ba-solutions (see details in the text). The profile of nerve terminal fluorescence is shown at the bottom. It is noticeable that the topographies of the spots are analogous (indicated by arrows) (B)

**Fig. 3:**
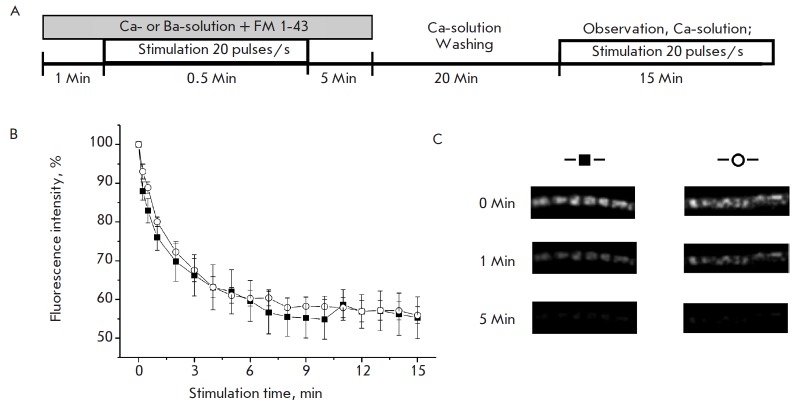
Exocytosis of synaptic vesicles loaded with the FM 1-43 dye in Ca- and Ba- solutions. Experimental scheme. Nerve terminals were loaded with the dye in Ca- and Ba- solutions and then unloaded in a Ca-solution (A). The dynamics of fluorescence intensity decay (dye unloading) of the nerve terminals preliminarily stained in Ca- (black squares) and Ba- (white circles) solutions, % of the initial value (B). Examples of fluorescent images from B (C)

The efficiency of synaptic vesicle endocytosis was evaluated by loading with a FM 1-43 dye under prolonged high-frequency stimulation in Ba- and Ca-solutions. It is known that the efficiency of capturing the FM 1-43 dye depends on the intensity of synaptic vesicle exocytosis and transmitter release. Therefore, the same number of transmitter quanta released during the stimulation time is needed to ensure loading with the FM 1-43 dye in Ba- and Ca-solutions. An analysis of the cumulative curves (the total number of synchronously and asynchronously released quanta, *Fig. 2A*) showed that 180,000 transmitter quanta in Ca-and Ba-solutions were released during stimulation for approximately 30 s. That is the duration of the stimulation we used to load the dye in our experiments. Under these conditions, we observed bright fluorescent spots in the Ca- and Ba-solutions (the fluorescence intensity of nerve endings was 0.114 ± 0.008 (*n *= 23) and 0.119 ± 0.011 relative units (*n *= 20), respectively) (*Fig. 2B*, *Fig. 3C*). These data indicate that during both synchronous and asynchronous vesicle exocytosis, effective recycling processes to form new synaptic vesicles occur.

The following experiments were aimed at assessing the ability of the vesicles involved in asynchronous transmitter release to undergo synchronous exocytosis.

For that purpose, nerve endings were preliminarily loaded with FM 1-43 in a Ba- or Ca-solution (stimulation of asynchronous or synchronous exocytosis, respectively) and the dynamics of dye-unloading under high frequency stimulation in a Ca-solution (synchronous exocytosis stimulation) was compared. It was shown that the dynamics of dye-unloading was the same (*Fig. 3B*) under these conditions. After 1 min of stimulation of the preliminarily loaded nerve endings in the Ba- and Ca-solutions, the fluorescence intensity decayed to 80.1 ± 1.2 (*n *= 7) and 76.0 ± 1.2% (*n *= 7), respectively; after 15 minutes, it decayed to 55.9 ± 2.2 (*n *= 7) and 55.3 ± 5.4% (*n *= 7), respectively, and fluorescent spots disappeared (*Fig. 3B*). Hence, the synaptic vesicles participating in asynchronous exocytosis and transmitter release were able to undergo synchronous exocytosis.

In several experiments we performed an in-depth analysis of the fluorescence spots in the nerve endings of the same sample preparation that occur during stimulation of synchronous and asynchronous release. First, the spots arising after dye-loading in a Ca-solution were analyzed. The dye was then unloaded (stimulation for 15 min); the nerve endings were re-loaded with the dye in the Ba-solution, and fluorescent spots were analyzed. A spatial analysis of the fluorescence spots of the same nerve endings in Ca- and Ba-solutions revealed their identity (*Fig. 2B*). These findings suggest that recycling processes occur in the same regions of the nerve endings adjacent to the active zones during both the synchronous and asynchronous vesicle exocytosis.

**Evaluation of participation of the recycling and reserve vesicle pools in asynchronous transmitter release**

**Fig. 4:**
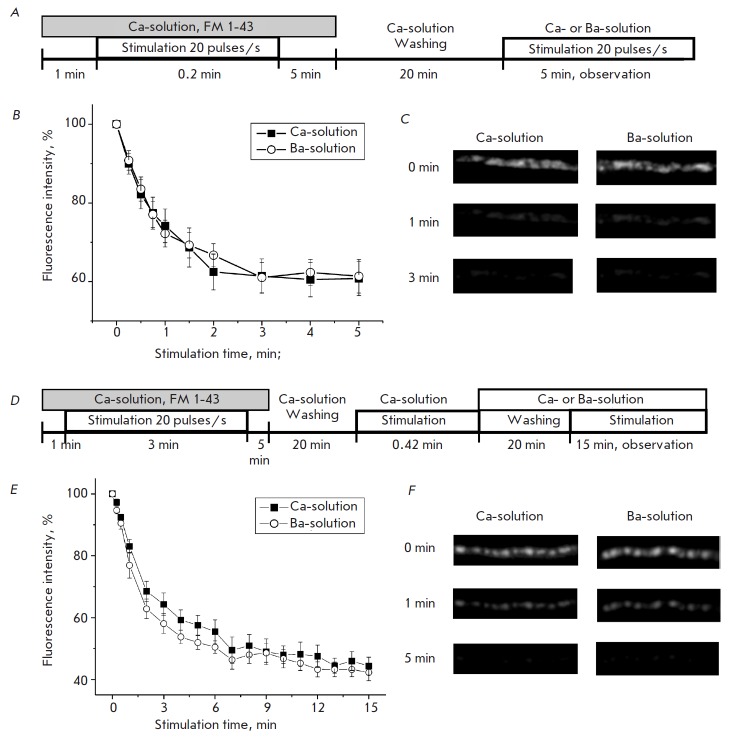
Exocytosis of the recycling and reserve synaptic vesicle pools in Ca- and Ba- solutions. Scheme of the experiment involving isolated staining and investigation of exocytosis of the recycling synaptic vesicle pool (A). The dynamics of fluorescence intensity decay of nerve terminals with apreliminarily stained recycling synaptic vesicle pool under highfrequency stimulation in Ca- (black squares) and Ba- (white circles) solutions (B). Fluorescence image examples from B (C). Scheme of the experiment involving isolated staining and investigation of exocytosis of the reserve synaptic vesicle pool (D). The dynamics of fluorescence intensity decay of nerve terminals with a preliminarily stained reserve synaptic vesicle pool under high-frequency stimulation in Ca- (black squares) and Ba- (white circles) solutions (E). Fluorescence image examples from E (F)

In this part of the study, isolated loading of either recycling or reserve vesicle pools with a FM 1-43 dye in a Ca-solution was conducted, followed by an evaluation of their ability to participate in asynchronous transmitter release. Short-term (12 s) high-frequency (20 pulses/s) stimulation was used for loading the recycling vesicle pool [26]. Weak fluorescent spots appeared in the nerve endings showing the accumulation of the recycling vesicle pool at active zones. Subsequently, we analyzed the dynamics of dye-unloading under stimulation of synchronous (Ca-solution) and asynchronous (Ba-solution) transmitter release. No differences in the fluorescence decay dynamics were revealed. After 1 min of stimulation in the Ca-and Ba-solutions, the fluorescence intensity of nerve terminals dropped to 74.2 ± 4.3 (*n *= 4) and 72.2 ± 3.4% (*n *= 5), respectively; after 5 min, to 60.8 ± 4.3 (*n *= 4) and 61.4 ± 4.3% (*n *= 5), respectively (*Figs. 4 B,C*).

The modified protocol was used for reserve vesicle pool loading [26]. Initially, the preparation was subjected to high-frequency stimulation for 3 min in a Casolution with FM 1-43. This protocol leads to staining of the recycling and reserve vesicle pools. After washing, the preparation was subjected to high-frequency stimulation again, but for 25 s, thus causing dye release by the recycling vesicle pool. As a result, the remaining stained synaptic vesicles in a nerve ending belonged mostly to the reserve pool [26]. The subsequent highfrequency stimulation of stained preparations in the Ca- (synchronous transmitter release stimulation) and Ba-solutions (asynchronous transmitter release stimulation) led to the same fluorescence decay in nerve endings (*Fig. 4E*). After stimulation in the Ca-and Basolutions for 1 min, the fluorescence intensity of nerve endings decayed to 83.1 ± 2.2 (*n *= 7) and 76.9 ± 4.1% (*n *= 6), respectively; after stimulation for 15 min, to 44.3 ± 2.9 (*n *= 7) and 42.3 ± 2.7% (*n *= 6), respectively (*Figs. 4E, F*). These data suggest that both the recycling and reserve vesicle pools are capable of asynchronous transmitter release, along with synchronous release.

## DISCUSSION

Experimental data showing differences in the mechanisms of synchronous and asynchronous transmitter release have been recently obtained. An assumption was made that both types of evoked transmitter release can be initiated in the region where various presynaptic calcium channels are located [9, 19, 27] using a multitude of protein molecules that provide the docking processes and synaptic vesicle fusion. Calcium-binding proteins synaptotagmins 1,2,9 are the main candidates for the role of a Ca-sensor of synchronous release; synaptotagmin 7 and Doc2 are the ones in asynchronous release [9]. The complexins and synaptobrevin 2 proteins are involved in the regulation of synchronous transmitter release; VAMP4 and synapsin 2 participate in the regulation of asynchronous transmitter release [28-26]. These findings raise the question of the identity of the vesicle pools involved in the synchronous and asynchronous transmitter release.

Our data show that the same synaptic vesicles are able to originate synchronous and asynchronous transmitter release with the same recycling processes. This is supported by the ability of the vesicles which have undergone the endocytosis-exocytosis cycle during synchronous transmitter release (Ca-solutions) to become involved in asynchronous exocytosis in Basolutions (*Fig. 4*). Conversely, the vesicles that are initially involved in asynchronous release (Ba-solutions) can be subsequently involved in synchronous release (Ca-solutions) (*Fig. 3 B,C*). Efficient dye entrapment under release stimulation in Ba-solutions (*Fig. 2B* *Fig. 3C*) indicates that both the asynchronous and synchronous transmitter release occur via full exocytosis of vesicles and subsequent formation of new vesicles by endocytosis. Synchronous and asynchronous exocytosis occur in the same areas of nerve endings at active zones, as evidenced by the complete identity of the topology and configuration of fluorescent spots under release stimulation in Ca-and Ba-solutions (*Fig. 2B*).

The ability of vesicles belonging to different functional pools of the nerve ending to become involved in asynchronous transmitter release was tested in experiments involving isolated staining of recycling and reserve vesicle pools. It was shown that the dynamics of dye-unloading from both the recycling and reserve vesicle pools in synchronous and asynchronous transmitter release is absolutely similar and that the fluorescence intensity decayed to the same level (*Fig. 4 B,E*). Hence, both vesicle pools equally participated in both the synchronous and asynchronous transmitter release. Our study did not confirm the view of some authors that the nerve endings may contain a separate population of vesicles that would trigger asynchronous transmitter release [18, 19] but not be involved in the evoked synchronous release.

## CONCLUSIONS

Our data suggest that the same synaptic vesicles originate both types of the evoked transmitter release in neuromuscular junction. It can be assumed that a synaptic vesicle contains the assembly of proteins required for both synchronous and asynchronous transmitter release. Probably the choice of the evoked release, in which the synaptic vesicle will participate, depends on the dynamics of the Ca^2+^ ions around the vesicles, vesicle arrangement with respect to a calcium channel, and the properties of the Ca-sensors of exocytosis.

## Abbreviations

EPP: end-plate potential
